# Evaluation of gender equality attitudes of Turkish and Foreign Nursing Students at a Private University

**DOI:** 10.12669/pjms.36.5.2365

**Published:** 2020

**Authors:** Serap Tekbas, Ganna Pola

**Affiliations:** 1 Dr. Serap Tekbas, ST., Department of Women Health, Near East University of Nursing Faculty, Lefkosa, Cyprus; 2 Dr. Ganna Pola, AP., Department of Women Health, Near East University of Nursing Faculty, Lefkosa, Cyprus

**Keywords:** Gender, Equality, Attitudes, Nursing Students

## Abstract

**Objective:**

To determine the gender equality attitudes of nursing students studying at a private university, the sociodemographic factors affecting gender equality, and the difference in gender attitudes between Turkish and foreign students.

**Methods:**

This cross-sectional study was carried out in the Faculty of Nursing at Near East University in March and April 2019. Three hundred six students studying in Turkish and English nursing programs in the thirdyear of study were included in the research. A sociodemographic questionnaire and the GEM scale were used as the data collection tools.

**Results:**

The mean age of the participants was 21.11 ± 3.88. 76.1% of the students were female and 33.3 students were foreign. The Gender Equality Men scores of the Turkish and foreign students were significantly different (p=0.001). Gender, the educational level of the father, a history of violence in the family, and talking about family and sexual issues affected the gender attitudes of all students.

**Conclusion:**

The results of the present study indicate that culture, gender, family education, and a history of violence in the family affected the gender equality scores of the nursing students.

## INTRODUCTION

The United Nations Development Program report (2016) stated that the Gender Equality Index for many undeveloped countries showed that inequality between the genders had increased. As a result, women were less educated, more exposed to violence, had less access to healthcare, and were poorer. According to this report, the gender equality is low in Sub-Saharan Africa.^1^,^2^ In research conducted by the World Economic Forum, gender inequality continues to grow even more. In this report, Turkey was ranked 131 among 140 countries in 2017.^3^,^4^

Gender equality refers to men and women having equal access to available resources, opportunities and power in areas including social institutions, family life, working life, legal regulations, education, politics, religion, and health; gender inequality refers to a situation in which one gender has more power and resources.^5^Culture is also an important factor in the formation of a gender equality.^6^ In recent years, much research has been carried out on this topic. These studies have shown that inequality directed against women is common worldwide. Factors such as family structures, education, the economy, religion, and social position affect how inequality emerges and is experienced .^7^

Nursing students are expected to develop an egalitarian attitude towards gender and to help shape the societies in which they are providing care.^8^ This study thus aimed to determine the attitudes towards gender equality of nursing students studying at a private university, the socio-demographic factors affecting gender equality, and the difference in perspectives towards gender between Turkish and foreign students.

## METHODS

This cross-sectional study was carried out in the Faculty of Nursing at Near East University in March and April 2019. Third-year students in the Turkish and English programs in the Faculty of Nursingwere included in the study. There were 204 students in the Turkish program and 102 (Nigerian=44; Zimbabwean=58) students in the English program. All third-year students in both programs were invited to participate in the study and 306 students took part. The reason third-year students were chosen is that they took a course on Women’s Health and Sexual Health in the first semester. This included the topics of gender equality, violence against women, and honor killing. As the students were due to graduate as nurses the following year later they would be using this information while they provided care. Ethical approval was obtained from the institution where the study was carried out (YDU/2019/66-752, dated 02.28.19). The data were collected using two forms.

### Sociodemographic Information

The researchers created a sociodemographic characteristics questionnaire with 16 questions. The participants were asked directly about their gender, age, nationality, the educational level of mother and father, economic status, employment status, marital status, family type and whether there was a family history of violence.

### Turkish and English Gender Equality Men (GEM) Scale

The GEM scale was originally developed in low income settings in Brazil and used as a tool to measure changes in gender-related interventions^9^ The GEM scale consists of items related to gender, domestic chores, violence, sexual relationships, masculinity, and sexual and reproductive behaviors. Each item is scored on a three-point Likert scale (“agree” =3, “somewhat agree” =2, “disagree” =1). The questionnaire has seven negative items that are reverse-scored and the total score obtainable ranges from 24 to 72. A higher score for the questionnaire means that the attitude of the individual towards gender equality is more positive and that the student is more inclined to support gender equality. The GEM scale includes two subscales: equitable gender norms and inequitable gender norms. The inequitable gender norms subscale has 17 items. For items one through17, the score for each item is based on three-point Likert scale (“agree” =3, “somewhat agree” =2, “disagree” =1). The minimum score obtainable from this subscale is 17 and the maximum score is 51. The equitable gender norms subscale has seven items. The minimum score obtainable from this subscale is 7 and the maximum score is 21. The total score from the scale is evaluated as high, medium or low: 1-23 points show low gender equality, 24-47 show medium gender equality, and 48-72 show high gender equality. The Form which was adopted by Ceber was used for the Turkish students.^10^ The English version of the same scale was applied to the foreign students.

### Analysis

For continuous data, the Mann-Whitney U test was applied for comparisons between two independent groups. The Kruskal-Wallis test was applied to compare continuous data among multiple groups. The LSD post hoc test (ANOVA) was used to provide information about which tools were significantly different from each other. The Cronbach’s alpha of the GEM scale was found to be 0.82. and the error rate was 5%. The significance level for all the studies was accepted as 0.05. Statistical evaluation of the data was performed using the SPSS 16.0 software.

## RESULTS

The mean age of the participants was 21.11 ± 3.88. 76.1% of the students were female and 33.3 students were foreigners 44.8% of the students’ fathers had primary school degrees, 49.5% of the mothers had the primary school degree.18.6% of the students defined their families as traditional. 58.8% of the students said that they can’t talk about a sexual issue with their family.

Turkish students’ 77.5%, foreign students’ 73.5% were female. 6.9% of Turkish students stated that there was violence in their families. This rate was 8.2% for foreign students. 38.2% of Turkish students and 47.1% of foreign students said that they could talk to their families about sexual matters.

When Turkish and foreign students’ GEM scale scores and subscale scores were compared in Table-I, total scale score and Inequitable Gender Norms were found to be significantly different (p<0.05). GEM scale total score (P=0.001) and the subscale score of foreign students were lower than Turkish students (P=0.001).

**Table-I:** The Comparison of Students’ Gender Equitable Men (GEM) Scale Score Points by Country (n=306).

Country	GEM Scale Total Score		Inequitable Gender Norms		Equitable Gender Norms	

	M±SD	Min-Max	M±SD	Min-Max	M±SD	Min-Max
Turkish Students(n=204)	54.358±7.715	30 - 72	45.074±6.955	19 - 51	9.284±3.707	7 - 21
Foreign Students(n=102)	51.118±4.629	41 - 66	42.137±4.88	29 - 51	8.980±3.707	7 - 21
*U	6336.00		6273.000		10088.500	
P	0.001		0.001		0.652	

* Mann-whitney test.

It was determined that there was a statistically significant correlation between the total GEM score averages of the foreign students and their age, fathers’ working condition, presence of domestic violence and state of talking about sexuality with their parents (p<0.05). (Table II). Examining the gender inequality subscale scores of the foreign students; the scores of the students who were under 20 years of age, were not able to talk about sexual issues with their parents and whose mother did not work and parents had primary school degree, were found to be lower than the scores of the students who were over 20 years of age, were able to talk about sexual issues with their parents and whose mother worked and parents had an educational degree higher than primary school. It was determined that there was no statistically significant correlation between the total GEM scale and subscale score averages and family type, working condition (p>0.05) (Table II).

**Table-II:** The Comparison of Foreign Students’ GEM Score and Subscore Points by Some Demographic Factor (n=102).

Foreign Students / caracterstic		GEM Total Score		Inequitable Norm		Equitable Norm	

		M±SD	P	M±SD	P	M±SD	P
Age	20 yrs & below (n=19)	49.32±3.59	*0.023	39.63±4.78	*0.005	9.68±3.32	0.344
	21yrs & above (n=83)	51.53±4.76		42.71±4.75		8.82±2.77	
Gender	Female (n= 75)	51.53±4.47	0.188	42.28±4.67	0.613	9.23±3.07	*0.020
	Male (n=27)	49.96±4.96		41.74±5.51		8.22±2.14	
Mother Work	Yes (n=74)	51.54±4.65	0.352	43.08±4.59	*0.001	10.36±3.26	*0.000
	No (n=28)	50.00±4.46		39.64±4.82		7.21±2.56	
Father Work	Yes (n=68)	50.38±4.86	*0.028	41.96±5.18	0.762	8.43±2.21	*0.003
	No (n=34)	52.59±3.79		42.50±4.27		10.08±3.68	
Family Type	Traditional (n=28)	49.82±3.63	0.086	41.54±3.99	0.520	8.29±1.86	0.304
	Modern (n=74)	51.61±4.89		42.36±5.19		9.24±3.15	
Job Status	Yes (n=6)	52.83±4.75	0.462	44.67±5.09	0.234	8.17±0.75	0.322
	No (n=96)	51.01±4.63		41.97±4.85		9.03±2.95	
Violence in the family	Yes (n=29)	51.93±7.22	*0.023	43.72±8.55	0.209	8.21±1.66	0.189
	No (n=175)	54.76±7.74		45.29±6.66		9.46±3.92	
Talking with parents about sexuality	Yes (n=78)	55.26±8.49	*0.002	45.44±7.86	*0.022	9.82±3.99	*0.043
	No (n=126)	53.80±7.18		44.85±6.36		8.95±3.49	
Mother’s Education	Primary education (n=3)	49.87±5.67	**0.217	39.65±4.85	**0.023	8.33±0.58	**0.689
	Secondary Education (n=23)	51.32±4.23		42.65±4.65		8.64±2.32	
	University Education (n=75)	54.67±4.93		46.33±5.51		10.22±4.22	
Father’s Education	İlliterate (n=6)	50.17±3.87	**0.781	37.13±3.87	**0.034	7.50±0.55	**0.005
	Primary education (n=8)	51.13±2.95		42.67±3.61		8.56±2.16	
	Secondary Education (n=20)	51.55±4.79		42.7±4.07		8.85±3.23	
	University Education (n=68)	51.07±43.86		42.51±5.04		14.00±3.74	
Economic Status	Poor (n=6)	51.83 ±3.31	**0.602	43.83±2.48	**0.460	8.00±0.89	**0.005
	Middle (n=61)	51.34±4.71		41.67±5.02		9.67±3.39	
	Good (n=35)	50.60±4.73		42.66±4.92		7.94±1.45	

* Mann-Whitney test,

** Kolmogorov smirnov test.

It was determined that there was a statistically significant correlation between the total GEM score averages of the Turkish students and their age, gender, fathers’ working condition, family type, working condition, presence of domestic violence, state of talking about sexuality with their parents and educational level of father (p<0.05). It was found that mother’s working condition, educational level of mother and the students’ economic condition had no impact on the total GEM scores (p>0.05) (Table III). Assessing the inequality norm subscale scores of the Turkish students; the scores were found to be lower in students who were male, employed, suffered from domestic violence, were not able to talk about sexual issues with their parents, had a good economic condition and whose father was unemployed and illiterate (Table III).

**Table-III:** The Comparison of Turkısh Students’ GEM Score and Subscore Points by Some Demographic Factor (n=204).

Turkısh Students caracterstic		GEM Total Score		Inequitable Norm		Equitable Norm	

		M±SD	P	M±SD	P	M±SD	P
Age	20yrs & below (n=19)	57.29±9.16	0.003	46.29±6.11	0.076	11±4.86	*0.001
	21yrs & above (n=83)	53.43±6.98		44.69±7.18		8.74±3.09	
Gender	Female (n= 75)	56.53±6.34	0.000	47.03±5.29	*0.0001	9.50±4.07	0.864
	Male (n=27)	46.89±7.40		38.35±7.79		8.54±2.09	
Mother Work	Yes (n=77)	55.79±8.93	0.379	45.52±6.58	0.931	10.27±4.97	0.020
	No (n=127)	53.49±6.76		44.80±7.18		8.69±2.51	
Father Work	Yes (n=160)	55.33±7.73	*0.0001	45.71±6.68	*0.004	9.61±4.06	0.104
	No (n=44)	50.84±6.62		42.75±7.51		8.09±1.39	
Family Type	Traditional (n=29)	51.93±7.22	*0.023	43.72±8.55	0.209	8.21±1.66	0.189
	Modern (n=175)	54.76±7.74		45.29±6.66		9.46±3.92	
Job Status	Yes (n=34)	51.41±7.48	*0.009	43.59±7.49	*0.034	7.82±0.67	*0.299
	No (n=170)	54.95±7.65		45.37±6.83		9.58±3.99	
Violence in the family	Yes (n= 14)	46.21±7.94	*0.0001	36.79±9.03	*0.0001	9.43±2.56	0.320
	No (n=190)	54.96±7.37		45.68±6.39		9.27±3.78	
Talking with parents about sexuality	Yes (n=78)	55.26±8.49	*0.002	45.44±7.86	*0.022	9.82±3.99	*0.043
	No (n=126)	53.80±7.18		44.85±6.36		8.95±3.49	
Mother’s Education	lliterate (n= 14)	53.92±9.71	**0.851	45.57±4.73	**0.207	8.43±2.28	**0.169
	Primary education (n=106)	54.00±4.49		45.62±6.33		8.88±3.13	
	Secondary Education (n=58)	54.50±6.93		44.64±8.19		9.74±4.51	
	University Education (n=26)	54.38±8.79		43.54±7.47		10.38±4.31	
Father’s Education	İlliterate (n=2)	40.00±5.66	**0.030	32.50±4.95	**0.001	7.50±0.71	**0.028
	Primary education (n=121)	52.29±8.05		41.94±7.77		8.67±3.04	
	Secondary Education (n=47)	54.00±10.89		43.83±8.90		10.17±4.86	
	University Education (n=34)	55.31±5.62		46.64±5.12		10.35±3.76	
Economic Status	Poor (n=38)	55.58±7.88	**0.217	45.89±7.32	**0.012	9.68±4.64	**0.0001
	Middle (n=126)	54.32±6.29		45.73±6.10		8.59±2.83	
	Good (n=40)	53.33±11.02		42.23±8.43		11.10±4.49	

* Mann-Whitney test,

** Kolmogorov smirnov test.

## DISCUSSION

In the present study, the total GEM score averages of the foreign students were found to be significantly lower than the Turkish students (p<0.05). Some socio-demographic features affecting the GEM scores of the Turkish and foreign students were different. The total GEM score averages were found to be significantly lower in the foreign students who were under 20 years of age and low in the Turkish students who were over 20 years of age. Also the total GEM score averages were found to be low in the Turkish students whose father was unemployed and significantly lower in the foreign students whose father was employed. In different studies it has been found that factors affecting gender equality attitudes, vary from society to society.^2^,^6^,^7^ In a study assessing gender attitudes of Latins and whites; it has been determined that Latins adopt a more traditional family structure, which considerably affects their gender role perception, sexual identity and power balance throughout their lives.^11^

Besides different reasons affecting the students’ GEM scores; gender, presence of domestic violence, being able to talk about sexual issues with parents and educational level of father decreased the total and / or subscale GEM score averages of all the students. In our study it was found that the equalitarian norm subscale score averages of the foreign male students were lower than the female students and the total GEM and inequality norm subscale score averages of the Turkish students were lower than the female students. The studies conducted in Turkey and Africa to determine gender roles of university students, have demonstrated that female students have a higher level of positive thinking regarding gender equality compared to male students.^5^,^12^-^14^ In another study conducted with medical students in Pakistan it was revealed that female students were exposed to gender discrimination more often.^15^

In our study the total GEM scale score averages were found to be lower in the Turkish (p=0.001) and foreign (p=0.023) students who suffered from domestic violence. In a study it was determined that equalitarian gender attitudes of girls who suffered from domestic violence, were affected negatively.^16^ The studies support the argument that domestic violence is an important risk factor for developing inequal gender attitude, which is in agreement with our study.^17^,^18^ In another study it was reported that being exposed to violence increased tendency to violence and sexist approach attitudes, which was associated with the fact that violence is a learned behavior and is handed down the next generations in this way.^19^ The reason for this matter is that children and youth take their parents as a model and the constantly encountered violence is perceived to be normal.

It was found that the Turkish and foreign students who were not able to talk about sexual issues with their parents, had significantly lower total GEM scale and subscale score averages. The studies have revealed that families who are open to communication concerning sexual issues, have more equalitarian gender norms regarding parenting roles.^20^,^21^ The reason for this condition is that equalitarian and conscious parents do not consider sexual issues a taboo and thus their children have an equalitarian gender attitude.

In our study one of the factors affecting attitudes toward gender roles was educational level of the parents of the students. It was found that as educational level of the parents of foreign students increased, their attitudes became more equalitarian. It was determined that the GEM scores of the Turkish students were affected by educational level of their father. It was observed that as educational level of the father of the Turkish students increased, they developed more equalitarian attitudes. In the study by Terzioglu it was found that as educational level of families increased, the gender equality perspective was affected positively, which is in agreement with our study.^22^

### Limitations of the study

As this study was conducted with the students who were enrolled only in the third-year nursing faculty of the related university, the results cannot be generalized to all students. All the data in relation to personality traits and attitudes toward gender roles were based on the personal statements, which requires considering the fallibility.

## CONCLUSION

The foreign students’ gender equality attitudes were found to be lower than the Turkish students, which was associated with cultural differences. It was determined that students who were male, suffered from domestic violence, were not able to talk about sexual issues with their parents, and whose father had a lower educational level, had lower gender equality attitudes. In order for the students to develop more equalitarian attitudes, it is important that they are taught again and again the significance of gender equality until graduation. The impact of culture on gender equality in educational planning, should be remembered.

### Authors Contribution

**ST:** Conceived, designed and did statistical analysis & editing of manuscript and takes the responsibility and is accountable for all aspects of the work in ensuring that questions related to the accuracy or integrity of any part of the work are appropriately investigated and resolved

**AP:** Did data collection and manuscript writing.

**ST:** Did review and final approval of manuscript.
